# Computer-Aided Diagnosis of Skin Lesions Using Conventional Digital Photography: A Reliability and Feasibility Study

**DOI:** 10.1371/journal.pone.0076212

**Published:** 2013-11-04

**Authors:** Wen-Yu Chang, Adam Huang, Chung-Yi Yang, Chien-Hung Lee, Yin-Chun Chen, Tian-Yau Wu, Gwo-Shing Chen

**Affiliations:** 1 Graduate Institute of Medicine, College of Medicine, Kaohsiung Medical University, Kaohsiung, Taiwan; 2 Department of Dermatology, E-Da Hospital, I-Shou University, Kaohsiung, Taiwan; 3 Research Center for Adaptive Data Analysis, Center for Biomarkers and Translational Medicine, and Graduate Institute of Biomedical Engineering, National Central University, Jhongli, Taiwan; 4 Department of Medical Imaging, National Taiwan University Hospital and National Taiwan University College of Medicine, Taipei, Taiwan; 5 Department of Public Health, Kaohsiung Medical University, Kaohsiung, Taiwan; 6 Department of Dermatology, Kaohsiung Municipal Ta-Tung Hospital, Kaohsiung, Taiwan; 7 Department of Dermatology, Kaohsiung Medical University Hospital, Kaohsiung Medical University, Kaohsiung, Taiwan; 8 Department of Dermatology, Kaohsiung Medical University Hospital. Department of Dermatology, College of Medicine, Kaohsiung Medical University, Kaohsiung, Taiwan; The University of Queensland, Australia

## Abstract

**Background:**

Computer-aided diagnosis (CADx) software that provides a second opinion has been widely used to assist physicians with various tasks. In dermatology, however, CADx has been mostly limited to melanoma or melanocytic skin cancer diagnosis. The frequency of non-melanocytic skin cancers and the accessibility of regular digital macrographs have raised interest in developing CADx for broader applications.

**Objectives:**

To investigate the feasibility of using CADx to diagnose both melanocytic and non-melanocytic skin lesions based on conventional digital photographic images.

**Methods:**

This study was approved by an institutional review board, and the requirement to obtain informed consent was waived. In total, 769 conventional photographs of melanocytic and non-melanocytic skin lesions were retrospectively reviewed and used to develop a CADx system. Conventional and new color-related image features were developed to classify the lesions as benign or malignant using support vector machines (SVMs). The performance of CADx was compared with that of dermatologists.

**Results:**

The clinicians' overall sensitivity, specificity, and accuracy were 83.33%, 85.88%, and 85.31%, respectively. New color correlation and principal component analysis (PCA) features improved the classification ability of the baseline CADx (p = 0.001). The estimated area under the receiver operating characteristic (ROC) curve (Az) of the proposed CADx system was 0.949, with a sensitivity and specificity of 85.63% and 87.65%, respectively, and a maximum accuracy of 90.64%.

**Conclusions:**

We have developed an effective CADx system to classify both melanocytic and non-melanocytic skin lesions using conventional digital macrographs. The system's performance was similar to that of dermatologists at our institute. Through improved feature extraction and SVM analysis, we found that conventional digital macrographs were feasible for providing useful information for CADx applications. The new color-related features significantly improved CADx applications for skin cancer.

## Introduction

Skin cancer is a commonly occurring malignancy in fair-skinned populations. In the last decade, the number of skin cancer treatments grew substantially, and the cost of skin cancer management was among the highest of all cancers in the United States [1]–[3]. There were approximately 76,250 new cases of melanoma and approximately 8,790 new melanoma-related deaths in 2012 in the United States [4]. Although the incidence rates of melanoma in Asians are lower than in Caucasians, non-melanoma skin cancers, such as squamous cell carcinoma (SCC) or basal cell carcinoma (BCC), contribute to significant morbidities among fairer-skinned Asians [5]. In a recent estimation by the Australian government, the total cost of diagnosing and treating non-melanoma skin cancer was 511 million Australian dollars in 2010 and will be 703 million in 2015 [6].

It is always important for clinicians to be able to recognize and accurately diagnose skin cancer in its early stages. When conducting a skin cancer screening, doctors usually identify suspect lesions by visual examination, which is highly dependent on specific training, and diagnostic accuracy can vary greatly among individuals with varied experiences [7]–[9]. In the U.K. and Australia, there has been increasing interest in improving the diagnostic performance of general practitioners in recognizing and accurately diagnosing skin cancers [10], [11]. With the development of computer-aided image analysis technologies, physicians may obtain an objective “second opinion” from computer-aided detection (CAD) or computer-aided diagnosis (CADx) software to refine their diagnoses [12]. In clinical practice, CAD has been widely used in the field of lesion detection, such as breast lesion detection in mammography [13]–[15], lung nodule detection on chest radiographs or CT scans [16]–[18], and polyp detection in CT colonography [19], [20]. CADx has also been applied to the analysis of nuclear medicine images [21], [22], skin lesions [23]–[26], and histopathological images [27]–[29]. CADx has been demonstrated to increase the diagnostic accuracy of trainees in the field of radiology. In dermatology, the benefits of the integration of CADx into the clinical diagnosis of pigmented skin lesions for dermatologists remain under investigation [26], [30], [31].

It has been suggested that the accuracy rate of clinicians can be improved with the support of dermatoscopy. However, this approach depends on specific training of a limited population of clinicians, and mainly dermatologic specialists who manage skin tumors [26]. Moreover, previous CADx studies in dermatology based on digitized color images or dermatoscopic images mainly focused on melanoma or melanocytic skin cancer detection [23], [32]–[36]. This approach is not generally applicable, especially given the low incidence of melanoma in Asians. We became interested in developing a diagnostic system that can also classify non-melanocytic skin cancers in Asian people. Considering easy accessibility to digital photography, the ability to analyze regular digital photographic images would be invaluable for general practitioners. This method could possibly play an important role in the remote analysis of skin lesions using digital photography for hospitals lacking dermatologic specialists.

Therefore, the purpose of this study was to investigate the potential for skin lesion classification by CADx utilizing regular digital photographic images. In particular, this study aimed to develop new color-related features for conventional photography by investigating multicolor channel characteristics using Pearson correlation coefficients and principal component analysis (PCA). The proposed CADx system was compared with conventional methods.

## Materials and Methods

### Ethics statement

This study was approved by the institutional review board of the Kaohsiung Medical University Hospital (KMUH-IRB-980433), and the requirement to obtain informed consent was waived.

### Data acquisition

Between January 2006 and July 2009, a total of 44418 digital photographs of consecutively biopsied or excised skin lesions for histological confirmation at the Department of Dermatology, Kaohsiung Medical University were taken by dermatologists for recording purposes prior to procedures. After removing images that were mis-registered or of poor quality (unfocused or containing a motion artifact), the database consists of 24178 images from 3964 subjects (4192 specimens). We retrospectively reviewed all cases and excluded nontumor specimens (N = 3415) or lesions that had undergone previous surgical procedures (N = 8). There were 676 subjects (769 specimens, 1899 images) remaining in the database. A dermatologist (W. Y. C., with 5 years of experience) carefully reviewed theses images and selected a representative close-up image for each lesion. Images with hair artifacts were not excluded because the pre-selection of data showed little influence in a large dataset in a previous study [23]. Our photography equipment was a 6.1-megapixel digital single-lens reflex camera (D70, Nikon Corporation, Tokyo, Japan) with an 18–50 mm F2.8 macro lens (Sigma Corporation, Fukushima, Japan). When obtaining close-up images, the target lesion was focused and located at the center of the photographs, and the size was controlled so as not to exceed 50% of the area of the photograph.

All of the lesions were observed at the clinic by board-certified staff dermatologists from our institute. The clinicians' impressions prior to biopsy were classified as “benign”, “malignant”, or “indeterminate”. When the clinicians were not confident enough to make a definite benign or malignant diagnosis, the clinical impression was considered as “indeterminate”. Histopathological diagnoses were used as a “gold standard” and were also classified as “benign” or “malignant”. A physician's classification was considered concordant if the category of primary diagnosis agreed with the final pathologic report. For example, the diagnoses were concordant for a clinically suspect BCC that was pathologically proven to be SCC because both tumors are in the malignant category.

### CADx system

**Figure 1:**
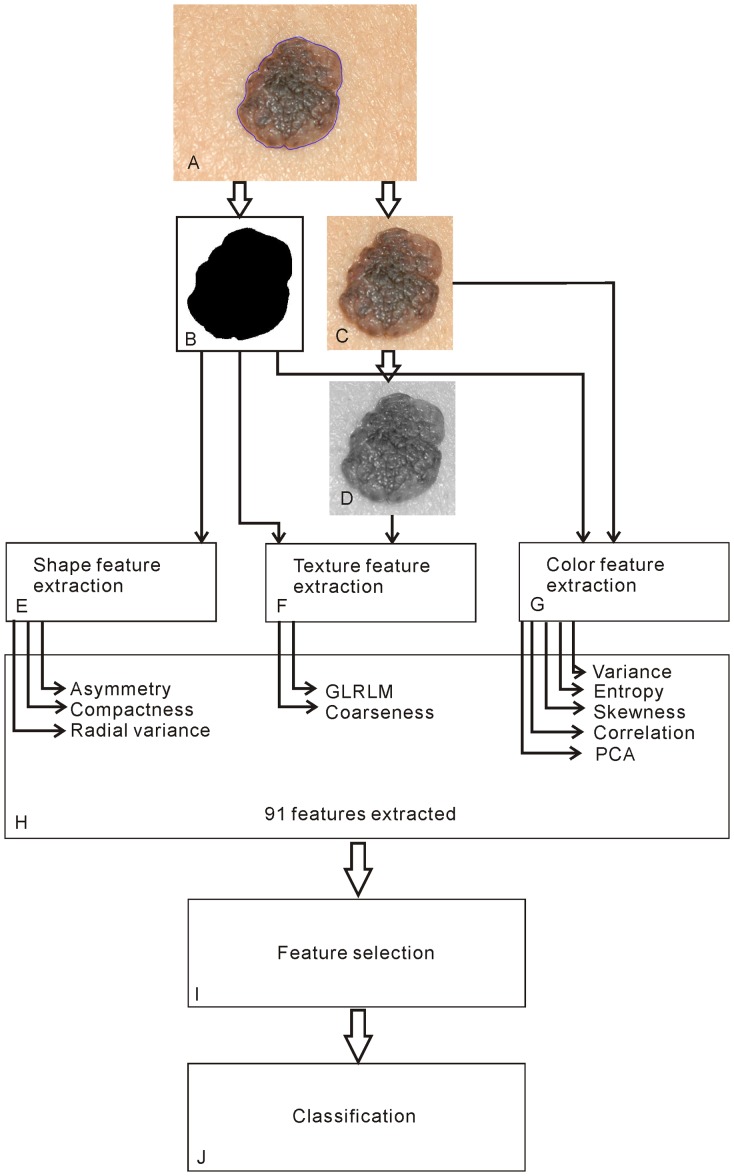
Flowchart of the CADx system.

A dermatologist (W. Y. C., with 5 years of experience) manually marked the borders of a skin lesion. The borders and their corresponding macrophotographs (Figure 1A) were then processed by a software system developed by our group. The software system consists of four components: image preprocessing, feature extraction, feature selection, and classification. These components are illustrated in Figure 1 and are explained in the following sections.

#### Image preprocessing

In previous studies, there were debates regarding the normalization of different lighting conditions and skin color using adjacent skin [37], [38]. In the current study, the original macrophotograph was cropped into two sub-images: one consisting of only lesion area and one consisting of a small rectangular sub-image that contained the entire targeted lesion plus a portion of the surrounding normal skin. To standardize this step, the cropped normal skin area was set equal to the lesion area, and the lesion was situated in the center of the sub-image (Figure 1C). A binary image mask (Figure 1B) was also derived to mark the lesion pixels.

#### Extracting conventional features

We investigated three groups (shape, texture, and color) of a total of 73 conventional features to build a baseline CADx. These features are briefly reviewed as follows.

The first group is shape features. From the binary image masks (Figure 1B), three conventional shape features were extracted: asymmetry, compactness, and radial variance [39], [40].

The second group is texture features. Two types of texture features were extracted: the gray-level run-length matrix (GLRLM) [41] and Tamura's coarseness features [42]. Tamura's coarseness estimates were computed for the entire sub-image (including both normal skin and the lesion) and for the lesion area alone. As GLRLM features were defined in rectangular images, these features were only computed for the entire sub-image. However, the grayscale images were converted from 256 to 16, 8, 4, and 2 levels of gray images. Eleven GLRLM features (listed in Table S1) were computed for each converted gray image. This computation resulted in two Tamura's coarseness and 44 GLRLM features.

The third group is conventional color features. Three common statistical features were considered: 1) variance, a measure of the spread of a dataset; 2) entropy, a measure of randomness; and 3) skewness, a measure of distribution asymmetry. These features were estimated for the gray-, red-, green-, and blue-channel values in the entire sub-image and in the lesion area. This analysis resulted in eight variance, eight entropy, and eight skewness features.

#### New color features

Two groups of a total of 18 new color features were investigated in this study to improve CADx performance. In contrast to the aforementioned conventional color features that only considered color data for one color channel at a time, these new color features were derived from multidimensional color channels simultaneously. The features are delineated as follows:

The first group is two-dimensional color features. The Pearson product-moment correlation coefficient is a measure of the linear dependence of two variables [43]. Figure S1A illustrates the correlation of red- and green-channel pixel values for the image in Figure 1C. Six correlation coefficients (red-green, green-blue, blue-red, red-grayscale, green-grayscale, and blue-grayscale) were computed for both the entire sub-image and the lesion area. This computation resulted in 12 correlation features.

The second group is three-dimensional PCA of color features. PCA is a linear transformation technique used to de-correlate data and maximize information content [44], [45]. Figure S1B illustrates an example PCA for the image in Figure 1C. The PCA technique basically analyzes an image's red, green, and blue (RGB) values to obtain a new coordinate system (Figure S1B), such that the greatest variance, known as the first principal component (PC1), lies on the first axis; the second principal component (PC2) is the greatest variance in a direction orthogonal to the first axis; and the third (PC3) is orthogonal to the first and second axes. Alternatively, the principal components PC1, PC2, and PC3 can also be estimated by projecting every pixel's RGB values onto the three principal axes to form individual histograms for computing the corresponding variances (Figures S1C–E). In this study, we computed all three principal components for both the entire sub-image and the lesion area. This computation resulted in six PCA color features.

#### Feature evaluation and selection

First, the receiver operating characteristic (ROC) method was used to evaluate each feature [46]. A univariate analysis based on the area under the ROC curve (Az) was performed to evaluate the 73 conventional and 18 new features individually. Second, multivariate analysis methods were used to remove less informative features, which provide less or duplicate information to differentiate malignancy, needed to be eliminated to yield a better, more compact subset of features for CADx optimization. In this study, we performed this selection task by utilizing a popular backward stepwise algorithm named recursive feature elimination (RFE) [47]. In summary, the algorithm removed the least informative features one at a time from a set of features by utilizing linear support vector machines (SVMs) [48]. The feature assigned the smallest weight by the SVMs was considered as the least informative. The elimination procedure was performed recursively until the set of remaining features was empty. The final ranking of features was based on the order of elimination. The RFE algorithm was applied to optimize both the baseline CADx, with a set of 73 (conventional) features, and the proposed CADx, with 91 features (73 conventional and 18 new features).

#### Software implementation

For feature selection, we used *mlpy* version 3.5.0, a freely available python library for machine learning [49], running on the Windows 7 (Microsoft, Richmond, WA, USA) platform. The linear classifier in *mlpy* was LIBLINEAR [50], and w^2^ was used for ranking criteria [47]. In-house CADx software for image processing was developed using MATLAB version R2012a (MathWorks, Natick, MA, USA) and the MATLAB code can be found in Compressed Archive S1.

### Statistics

Using pathological results as a gold standard, the sensitivity and specificity of classifying skin lesions by individual and all dermatologists were assessed. The performance of discrimination between malignant and non-malignant lesions by CADx was evaluated using leave-one-out cross-validation (LOOCV) with ROC curve analysis. The AUC of the ROC (Az) was estimated for every set of the top-*n* feature set ranked by the RFE algorithm. Thus, Az was estimated for the baseline CADx using the top 1, top 2, to top 73 conventional features and for the proposed CADx using the top 1 to top 91 of all features. The performance difference between the baseline and the proposed CADx was estimated using a paired bootstrap t-test based on the Az estimated for the optimal sets of features selected by RFE. The Az values of two ROC curves were compared using DeLong's test [51]. Finally, we compared the diagnostic accuracy of CADx with that of individual and overall clinical diagnoses for malignant skin lesions. All statistical analyses were performed using R version 3.0.0 [52]–[54].

## Results

### Demographics

A total of 769 images of distinct regions of interest (ROI) were obtained from 676 patients, including 296 males (43.8%) and 380 females (56.2%) with a mean ± SD age of 47.6±21.0 years. These images included 174 malignant lesions and 595 benign lesions. The demographic data for each histological diagnosis are summarized in Table 1. There were eight melanomas in this study, including four invasive melanomas and four noninvasive melanomas. The number of melanomas is small in our database, consistent with the relatively low incidence in the Asian population compared with Caucasians.

**Table 1:** Demographic data for each histological diagnosis and the performance of dermatologists and CADx.

					No. of Correct/Incorrect Diagnoses^2^	
Pathology	N^1^	%	Sex (F/M)	Mean Age (Year)	Dermatologist	CADx
**Benign**	595	77.37	358/237	40.88	511/84	521/74
Blue nevus	22	2.86	16/6	35.64	19/3	20/2
Compound nevus	53	6.89	35/18	28.68	50/3	47/6
Congenital nevus	9	1.17	3/6	22.89	9/0	6/3
Dermatofibroma*	49	6.37	30/19	36.84	47/2	48/1
Dysplastic nevus	2	0.26	1/1	39.5	1/1	1/1
Epidermal nevus*	3	0.39	2/1	17	3/0	2/1
Hemangioma*	38	4.94	22/16	47.21	35/3	23/15
Intradermal nevus	240	31.21	161/79	36.98	209/31	225/15
Junctional nevus	46	5.98	29/17	35.85	39/7	42/4
Lentigo simplex	1	0.13	0/1	72	0/1	1/0
Nevus lipomatosus superficialis*	1	0.13	0/1	57	1/0	0/1
Nevus sebaceous*	2	0.26	2/0	30.5	2/0	2/0
Nevus spilus	2	0.26	2/0	23.5	2/0	2/0
Seborrheic keratosis*	127	16.51	55/72	57.69	94/33	102/25
**Malignant**	174	22.63	81/93	68.86	145/29	149/25
BCC*	110	14.3	57/53	68.14	97/13	99/11
Cutaneous melanoma	8	1.04	5/3	63.25	6/2	6/2
Kaposi's sarcoma*	14	1.82	2/12	70.5	11/3	10/4
Keratoacanthoma*	22	2.86	7/15	64.82	14/8	18/4
SCC*	20	2.6	10/10	78.35	17/3	16/4
**All lesions**	769	100	439/330	47.21	656/113	670/99

1 N: number of images.

2 Indeterminate diagnoses by dermatologists were considered as incorrect. The diagnoses by CADx were made using the final 16-feature model with a cutoff value 0.3972.

* Non-melanocytic skin lesions.

### Clinical performance

**Figure 2:**
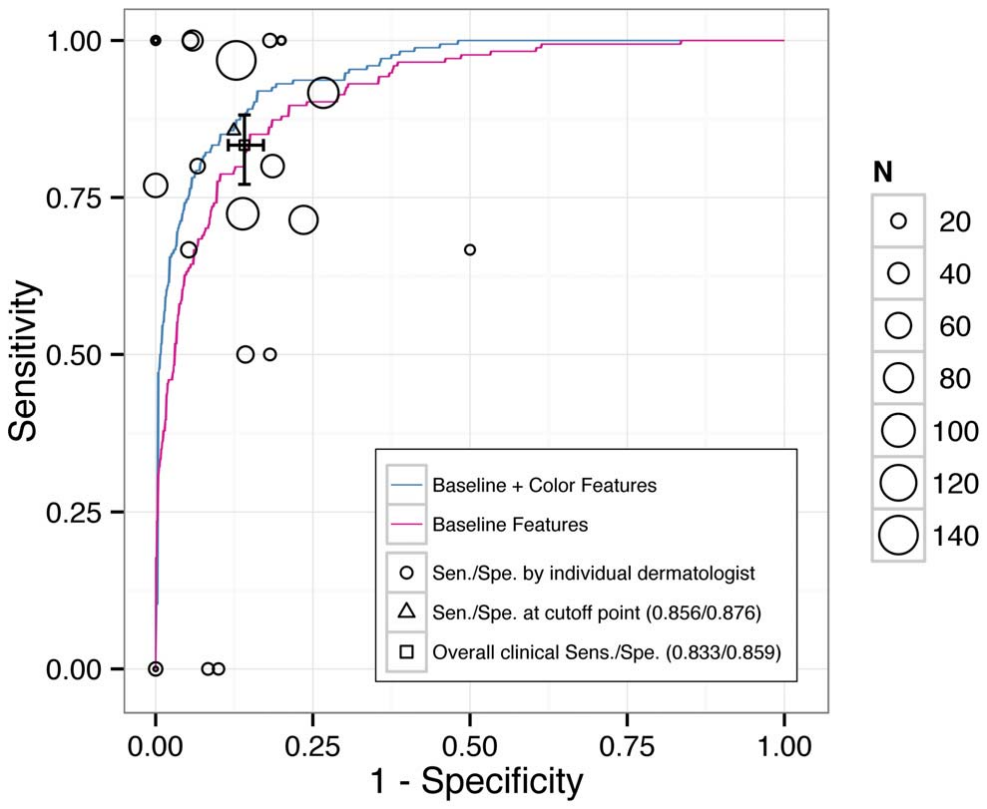
Comparison of the diagnostic performance of CADx systems and dermatologists. The ROC curves for differentiating between benign and malignant lesions using the baseline (red line) and proposed (blue line) CADx systems. The clinical sensitivity and specificity of the performance of the dermatologists (circles) at our institute are shown, and the area of the circle indicates the number of biopsies performed by each doctor. Note that the clinical sensitivity and specificity were calculated presuming that the clinician always made a “wrong” diagnosis for “indeterminate” lesions.

Face-to-face clinical diagnoses of skin lesions were made by 25 staff dermatologists at our institute. Clinical performance determined based on lesions about which the physicians were more confident and for which they could provide a definite diagnosis would be better than the true performance of clinicians, assessed by including lesions about which they were less confident or which they were even unable to discriminate as benign or malignant. To prevent selection bias and the overestimation of clinical performance, indeterminate cases were not removed and were considered “incorrect” in this study because the clinician was unable to make a correct diagnosis. In the 769 lesions, there were 74 (15 malignant and 59 benign) clinically indeterminate lesions. By categorizing these cases as incorrect diagnoses, the overall sensitivity, specificity, and accuracy were 83.33% (95% confidence interval (CI), 77.09–88.14%) (145/174), 85.88% (82.85–88.45%) (511/595), and 85.31% (82.63–87.63%) (656/769), respectively. The performance of each dermatologist is illustrated in Table 1 and Figure 2.

### CADx performance

The Az values of 91 studied features, ranging from 0.503–0.823 and determined by univariate analysis, are summarized in Table S1. The higher the Az, the better that a feature performs individually. Compactness achieved the highest Az (0.745) among the shape features. Texture features scored moderately, from 0.511–0.769. Conventional color features apparently scored below average, from 0.506–0.703. In comparison, new correlation and PCA color features achieved a much higher Az, from 0.504–0.823.

**Figure 3:**
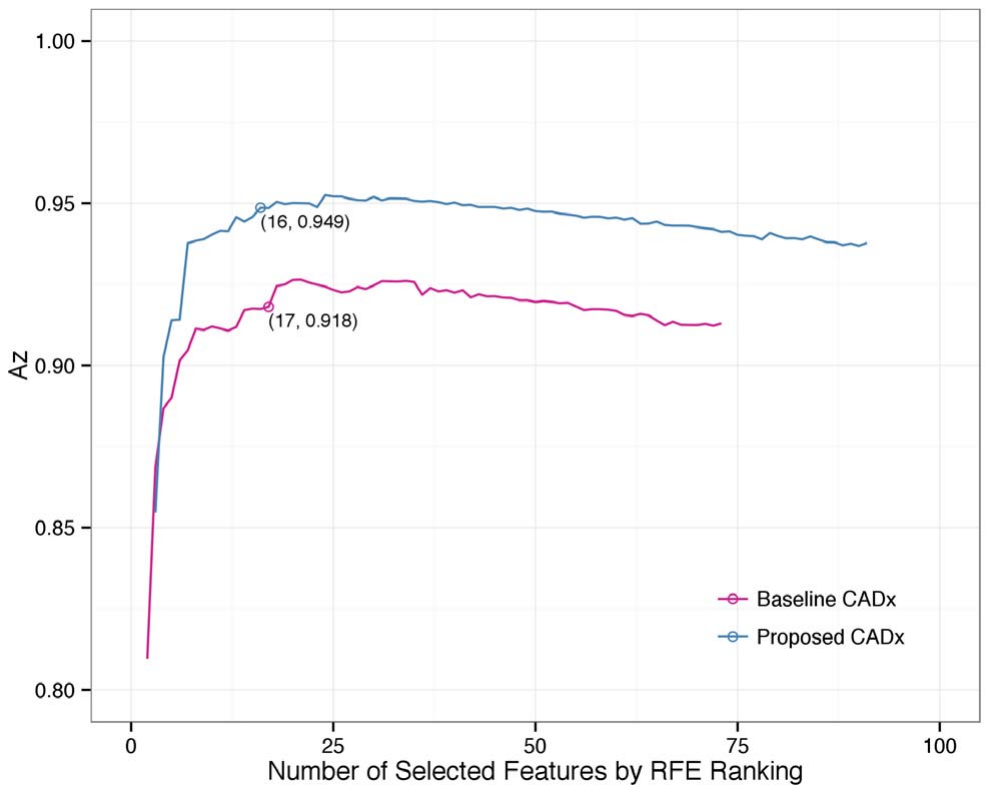
Az with different numbers of features for the baseline and proposed CADx systems. After adding new color-related features, the proposed CADx had a better Az performance than the baseline CADx system did.

The ranking of 73 conventional and all 91 (plus 18 new color-related) features, determined using the RFE algorithm for feature selection in the multivariate analysis, is also summarized in Table S1. The higher the ranking, the more discriminating information that a feature contributes collaboratively. Four new color features were ranked in the top 10 of all 91 features. These features were the PC3 of the lesion area alone and for the entire cropped image (ranked first and eighth) and the green-blue and green-grayscale correlation coefficients of the lesion area alone (ranked sixth and seventh). The optimal operating Az was 0.953 (95% CI, 0.934–0.968) by the new CADx using 24 features compared with 0.927 (95% CI, 0.906–0.947) by the baseline CADx using 21 features. The Az improved significantly, by 0.026 (p = 0.001). To avoid over-fitting, the variable number in the final model was reduced to be less than one tenth of the number of malignant lesions (N = 174) [55], [56]. After reducing the feature number, the optimal operating Az was 0.949 (95% CI, 0.932–0.965) by the new CADx using 16 features compared with 0.918 (95% CI, 0.895–0.941) by the baseline CADx using 17 features. The Az improved significantly, by 0.031 (p = 0.001). Figure 3 illustrates the Az of the baseline and proposed CADx for all possible top-*n* selected features.

**Figure 4:**
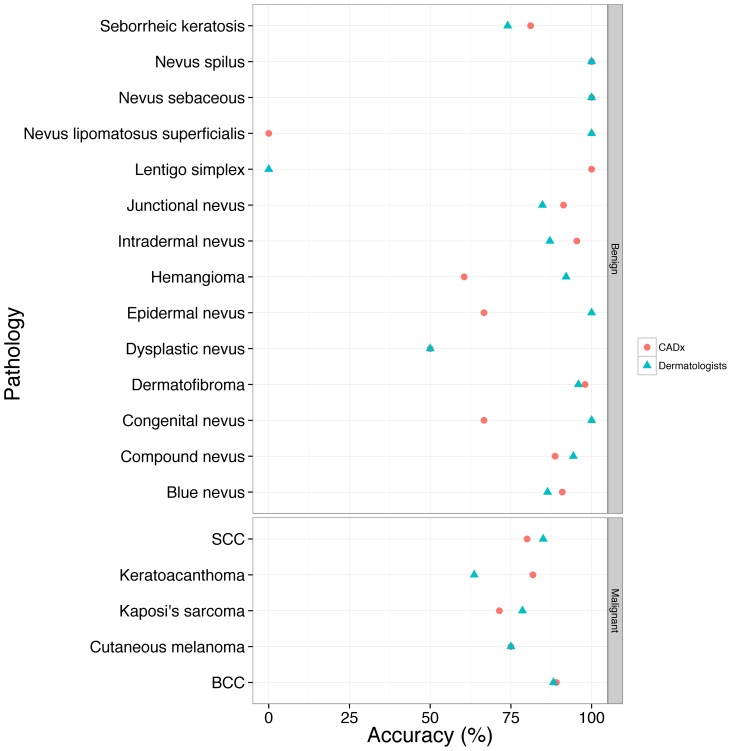
The accuracy of dermatologists and CADx for different pathological diagnoses.

**Figure 5:**
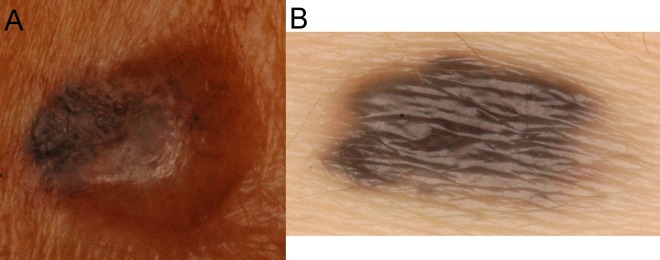
Two lesions with incorrect clinical diagnoses but correct CADx categorization. Two lesions with incorrect clinical diagnoses but correct CAD system categorization. (A) Basal cell carcinoma. A skin nodule with variegated color. The clinical impression was a benign epidermal cyst. (B) Intradermal nevus. An asymmetric pigmented nodule with an irregular border. The clinical impression was malignant melanoma.

To compare the performance of CADx and dermatologists, the two best feature sets with the largest Az from the baseline and proposed CADx were chosen. Figure 2 presents the performance of the discrimination of skin malignancy by the baseline and proposed CADx and the individual and overall sensitivity/specificity of clinical diagnosis by dermatologists. The accuracy of clinicians and CADx regarding each pathological diagnosis is shown in Figure 4. The proposed CADx system (using the top 16 features) performed comparably to the overall performance of the dermatologists. The maximal accuracy was 90.64%, at which the sensitivity and specificity were 78.16% and 94.29%, respectively. By adjusting the operating point to be similar to that of dermatologists, CADx sensitivity and specificity were 85.63% and 87.65%, respectively. These numbers were similar to the overall clinical sensitivity (83%) and specificity (85.88%) determined for all dermatologists. After grouping the lesions into melanocytic (N = 383) or non-melanocytic (N = 386) lesions, the diagnostic accuracy of the proposed CADx was 91.38% and 82.90%, respectively. Two examples of skin lesions with incorrect clinical diagnoses but correct CADx classification are shown in Figure 5.

## Discussion

In this study, a prototype of a CADx system was developed for the classification of skin lesions using a state-of-art machine learning technique. Whereas the baseline CADx system uses features that dermatologists usually consider in clinical practice, the introduction of new color-related features increases the Az of the baseline CADx system. The strength of this study lies in its generous inclusion criteria and good-sized dataset, which represents a wide spectrum of skin lesions encountered in daily practice, with each lesion given a definite histopathological diagnosis. Our classification system was developed based on substantially heterogeneous lesion categories and aimed to identify features of digitized images of skin lesions, which had not been developed before for Asians.

In this study, we investigated the potential diagnostic value of digital macrophotography and made two important findings. First, by taking advantage of a good-sized dataset of heterogeneous skin lesions, we found that useful information can be extracted from clinical macrophotographs, which are recorded by a regular digital camera, to help to classify malignancies using modern machine learning technologies such as SVM and RFE. By extracting only the conventional shape, color, and texture features, the baseline CADx achieved a high Az performance (0.918; 95% CI, 0.896–0.941), where 0.8< Az ≤0.9 is considered as good performance, and 0.9< Az ≤1 is considered as excellent. Second, we found that multidimensional color features such as correlation and PCA significantly improved CADx (p = 0.001) to a maximal Az of 0.949 (95% CI, 0.932–0.965). Four of the 18 investigated new color features (the PC3 of the lesion alone, the PC3 of the entire cropped image, and the green-blue and green-gray correlation for the lesion area alone) were ranked by RFE in the top 10 of all 91 studied features (Table S1). The high ranking indicates that the multidimensional approach generated good color features. More importantly, the significant Az improvement by 0.031 indicates that extra discriminating information of color variance was uncovered by adding new features. The clinical performance of the diagnosis of skin lesions varies within different institutes, and at our hospital, the clinical performance of the dermatologists varied widely as well (Figure 2), which may be due to the different experience levels of the clinicians and the complexity of the cases [23], [57]. Therefore, we compared CADx with the overall performance of the dermatologists and found that the diagnoses were similar.

Color variance has been proposed to be an important feature in clinical observation by both the naked eye and dermatoscopic examination. In the ABCD rule, the number of colors is positively related to the risk of malignancy. However, these color impressions are mainly subjective descriptions that remain extremely difficult to quantify. This difficulty is rooted in complex human color perception mechanisms. Cone cells in the human retina are three different types of light-sensitive photoreceptor cells that peak at RGB colors and can receive signals by responding to visible colors to different degrees. The human brain perceives colors through an opponent process of color vision by detecting differences between the three cone cell types, allowing humans to perceive different colors. Although the human brain is good at color recognition for discriminating a lesion from normal skin, the brain is not good at the quantification of perceived color variance within lesions. With a filter array of primary colors, the RGB components can be extracted from different colors by a camera and can be stored as digital data, which in turn can be quantified and analyzed by CADx. The variance of the blue channel, for example, was ranked in the top 3 (Table S1) in both the baseline and the proposed CADx systems. This concept suggests that the quantification of single-color channels by the CADx system provides important diagnostic values for baseline features.

When clinicians evaluate color variance, related criteria, such as pigmentary change, secondary ulceration, and abnormal vascular growth, significantly contribute to decision making. Because each lesion has a different RGB distribution in the color space, the conventional color features defined in the pure red, green, and blue channels may not capture the entire spectrum of all colors under consideration. Furthermore, the statistics of pixel RGB values could be affected by artifacts, such as reflection of the corneal layer, which may shift the actual color toward brightness, or the shadow of steric information, which may shift the actual color toward darkness in conventional photography. Digital dermatoscopic systems, which decrease reflection of the corneal layer, have been preferred over conventional digital photography in recent CADx research studies [23], [58], [59]. To overcome the aforementioned problems, we investigated two multidimensional color analytic approaches: correlation and PCA. As far as we know, multidimensional color features have not been reported as potent features for skin cancer analysis and diagnosis. Our findings suggest that multidimensional analysis could be a useful tool for quantifying skin color variance in digital macrophotography.

Correlation coefficients examine the linear relationship between two color channels or between one color and grayscale. The results indicated that the color correlation features derived from the entire cropped image achieved high Az values, between 0.508 and 0.823, compared with conventional color features' Az values, which were between 0.503 and 0.703. The green-blue and green-grayscale correlation of the entire cropped image were ranked as the sixth and seventh features by RFE, whereas the green-channel variance of the lesion area alone dropped from fifth to fifty-ninth. By considering PCA in terms of geometry, three principal components reflect the three-dimensional “shape” of the distribution of pixels in the RGB space. For example, if a pigmented BCC presents with ulceration and telangiectasia, the three-dimensional “shape” of the distribution might be more dispersed than a benign nevus, which often consists of mainly a plain brownish color. Among all PCA features, the PC3 derived from both the lesion area and the entire cropped image achieved the best Az. Variance along three principal color components of the lesion area was eliminated early during feature elimination, and the PC3 of both the lesion area alone and the entire cropped image were highly ranked as first and eighth. This finding might be explained by the following. The color distribution of the lesion or skin alone is more similar to an oval or spindle shape (Figure S1B), and each would not produce a specific pattern of malignancy. The skin color could provide a baseline, whereas inclusion of the lesion would change the shape of the color distribution being analyzed. The mixture of these two regions introduces the irregularity of the color shape in the color space. The PC1 coordinate roughly reflects the direction of luminance and is therefore not a good discriminating feature. PC2 and PC3 are the diagonal directions regarding the largest variance of colors, which might be affected by the variance of the lesion and skin themselves and/or by the difference between the lesion and the skin. Through the process of RFE, the high ranking of PC3 suggests that this component may be a new discriminating feature in the diagnosis of malignant skin lesions. After de-correlating the colors, the CADx system helps to analyze and quantify the three principal components of each lesion, thus aiding diagnostic accuracy, whereas human or conventional 1D color features have little use in the quantification and analysis of this type of color variance.

The necessity of normalization using adjacent normal skin has been debated [37], [38]. In the present study, all color features were extracted from images with and without the inclusion of adjacent normal skin. Including the adjacent skin area did improve the Az performance of all multidimensional color features except PC1. The exact mechanism of how the inclusion of normal skin area aids these features performance requires further study. The use of the CADx system for analyzing the complex color space might compensate for the weakness of human eyes and help to diagnose malignant lesions.

Regarding texture analysis, Tamura's coarseness was selected because it corresponds well to human visual perception of spatial variation of grey levels. The coarseness procedure estimates the differences between the averages of neighbor blocks of various sizes. The larger the block size generating the maximal difference, the coarser the image texture is. GLRLM features were selected for a similar visual perception reason. Consecutive pixels along a selected orientation tend to have the same intensity in a smooth region (with a long run length) while their values change significantly in rough regions (with a short run length).

In clinical settings, sensitivity and specificity varied widely between doctors at our institute (Figure 2). The result is compatible with previous reports stating that diagnostic performance is related to individual clinical experience and specific training in managing skin lesions [9], [57]. The proposed CADx system is not intended to replace routine clinical examinations, but with a high Az (0.949) and a manageable size of 16 meaningful features, the system may have a role in providing consistent criteria for feature measurement by on-site computation, a stable second opinion for less-experienced staff, or support for a clinical decision. Additionally, CADx may provide rapid second opinions in the setting of diagnosis by teledermatology, especially in a clinical situation in which it is not easy to access an expert clinician.

Several limitations of this study need to be acknowledged. The feasibility relies on a single image-capture system and consistent quality control for each image. To use a histopathological report as a gold standard, the analysis must be restricted to biopsied lesions. Those lesions for which clinicians or patients decided not to perform biopsy were not included in the dataset. All images in the dataset in our study were from Asian patients visiting a single center in southern Taiwan, so we do not know the system's performance with skin types of different races. Therefore, the generalizability of our results may be limited to biopsied lesions in Asians. There is concern about a single dermatologist drawing the lesion margin because the accuracy of the study might be compromised due to human subjectivity [24], [60]. Research on margin drawing by a group of experts or on the development of automated lesion segmentation should be performed in future studies.

## Conclusion

In conclusion, we have developed an effective CADx system that has performance similar to that of the dermatologists at our institute and that classifies both melanocytic and non-melanocytic skin lesions by utilizing conventional digital macrophotographs. Through advanced feature selection and SVM analysis, we also found that the new color correlation and PCA features significantly improved CADx applications for skin cancer.

## Supporting Information

Figure S1 **Correlation of red- and green-channel pixel values of skin lesions and PCA analysis of the lesion image.** (A) It shows that the lesion area (blue cloud points) has a wider width (indicating higher color variation) and therefore, has a lower correlation coefficient value than the normal skin area (red cloud points). Six correlation coefficients (red-green, green-blue, blue-red, red-grey, green-grey, and blue-grey) were computed for both the whole sub-image and the lesion area respectively. (B) Three new coordinate axes computed by using PCA (blue: lesion pixels; red: normal skin). (C, D, E) Histograms of the lesion (blue) and normal skin (red) for projected data on the directions of the first, second and third principal components respectively.
(TIF)

Compressed Archive S1 **MATLAB code of the software that was used for feature extraction and classification.**
(ZIP)

Table S1 **The area under receiver operator characteristic curve (Az) of different features using univariate analysis and their ranking after recursive feature elimination (RFE) procedure using SVM.**
(DOC)
