# Supplementary figures and images for: Computer-Aided Diagnosis of Skin Lesions Using Conventional Digital Photography: A Reliability and Feasibility Study
Source: PLoS One. 2013 Nov 4;8(11):e76212. doi: 10.1371/journal.pone.0076212 (PMC3817186; doi:10.1371/journal.pone.0076212)

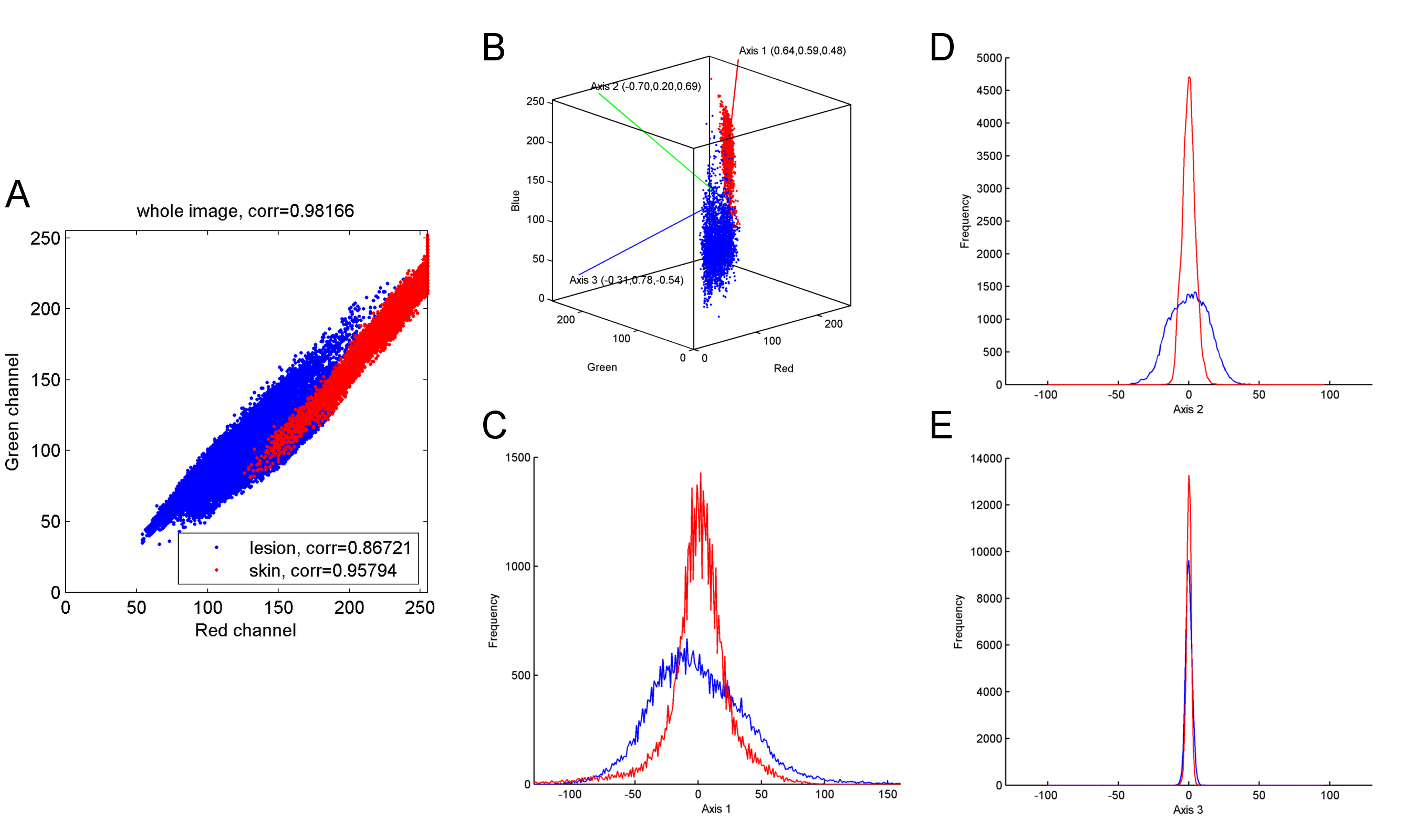

Supplement: Figure S1 — Correlation of red- and green-channel pixel values of skin lesions and PCA analysis of the lesion image. (A) It shows that the lesion area (blue cloud points) has a wider width (indicating higher color variation) and therefore, has a lower correlation coefficient value than the normal skin area (red cloud points). Six correlation coefficients (red-green, green-blue, blue-red, red-grey, green-grey, and blue-grey) were computed for both the whole sub-image and the lesion area respectively. (B) Three new coordinate axes computed by using PCA (blue: lesion pixels; red: normal skin). (C, D, E) Histograms of the lesion (blue) and normal skin (red) for projected data on the directions of the first, second and third principal components respectively. (TIF) [file pone.0076212.s001.tif]

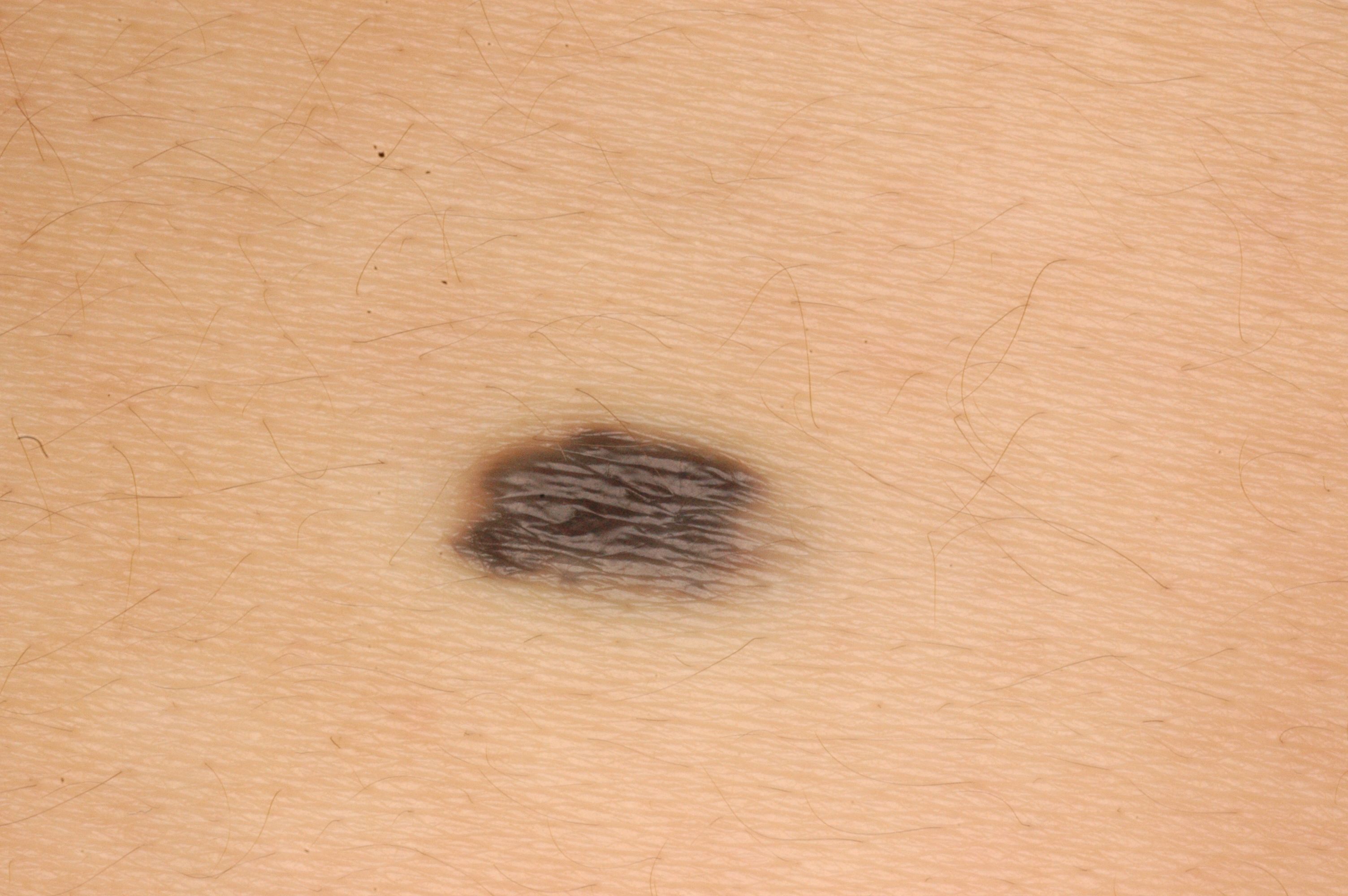

Supplement: Compressed Archive S1 — MATLAB code of the software that was used for feature extraction and classification. (ZIP) [file pone.0076212.s002.zip › SkinCad/data/2008-1039.jpg]

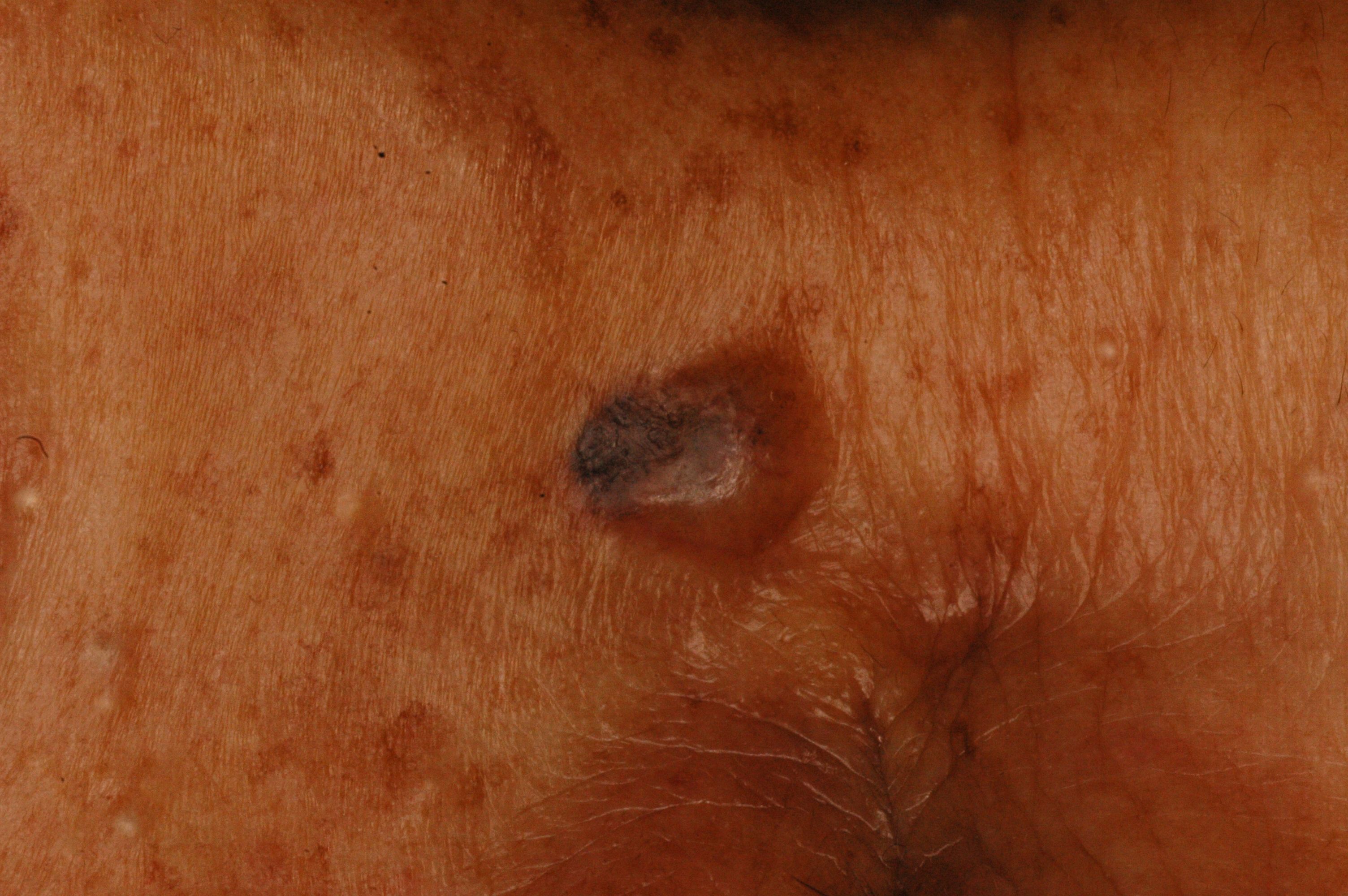

Supplement: Compressed Archive S1 — MATLAB code of the software that was used for feature extraction and classification. (ZIP) [file pone.0076212.s002.zip › SkinCad/data/2008-994 (1).jpg]
