# Supplementary material for: Computer-Aided Diagnosis of Skin Lesions Using Conventional Digital Photography: A Reliability and Feasibility Study
Source: PLoS One. 2013 Nov 4;8(11):e76212. doi: 10.1371/journal.pone.0076212 (PMC3817186; doi:10.1371/journal.pone.0076212)
Supplement: Table S1 — The area under receiver operator characteristic curve (Az) of different features using univariate analysis and their ranking after recursive feature elimination (RFE) procedure using SVM. (DOC) [file pone.0076212.s003.doc]

Table S1. The area under receiver operator characteristic curve (Az) of different features using univariate analysis and their ranking after recursive feature elimination (RFE) procedure using SVM.

| **Shape Features** | | | | | | | | | **Tamura’s Coarseness Features** | | | | | | | | |
| --- | --- | --- | --- | --- | --- | --- | --- | --- | --- | --- | --- | --- | --- | --- | --- | --- | --- |
| Asymmetry | | | | 0.688^a^/60^b^/61^c^ | | | | |  | Whole Image | | | | 0.608/38/63 | | | |
| Compactness | | | | 0.745/3/4 | | | | |  | Lesion-only | | | | 0.597/14/16 | | | |
| Radial variance | | | | 0.579/4/5 | | | | |  |  | | | |  | | | |
| **GLRLM Features** | | | | | | | | | | | | | | | | | |
|  | 2-GL | | | | | 4-GL | | | | | | 8-GL | | | | 16-GL | |
| SRE | 0.713/46/48 | | | | | 0.676/61/89 | | | | | | 0.669/67/81 | | | | 0.644/8/22 | |
| LRE | 0.676/58/68 | | | | | 0.600/37/44 | | | | | | 0.600/50/45 | | | | 0.589/72/86 | |
| LGRE | 0.769/59/76 | | | | | 0.565/29/46 | | | | | | 0.538/64/84 | | | | 0.544/18/24 | |
| HGRE | 0.769/48/62 | | | | | 0.661/53/13 | | | | | | 0.656/56/52 | | | | 0.659/73/72 | |
| SRLGE | 0.688/47/85 | | | | | 0.673/57/14 | | | | | | 0.645/44/73 | | | | 0.620/51/71 | |
| SRHGE | 0.717/7/15 | | | | | 0.596/54/79 | | | | | | 0.577/34/26 | | | | 0.527/27/21 | |
| LRLGE | 0.619/69/91 | | | | | 0.548/65/87 | | | | | | 0.531/36/23 | | | | 0.511/40/70 | |
| LRHGE | 0.727/6/40 | | | | | 0.675/35/25 | | | | | | 0.681/70/78 | | | | 0.683/52/88 | |
| GLNU | 0.689/49/66 | | | | | 0.675/71/77 | | | | | | 0.696/12/64 | | | | 0.693/33/83 | |
| RLNU | 0.716/25/90 | | | | | 0.654/28/42 | | | | | | 0.650/55/43 | | | | 0.632/11/51 | |
| RPC | 0.691/26/67 | | | | | 0.622/32/41 | | | | | | 0.625/13/19 | | | | 0.612/23/20 | |
| **Single Color Channel Features** | | | | | | | | | | | | | | | | | |
|  | Red-channel | | | | | Green-channel | | | | | | Blue-channel | | | | Gray-level | |
| Variance I^1^ | 0.597/42/56 | | | | | 0.691/5/59 | | | | | | 0.703/1/2 | | | | 0.670/17/60 | |
| Variance II^2^ | 0.507/31/28 | | | | | 0.504/21/27 | | | | | | 0.517/2/3 | | | | 0.508/22/29 | |
| Entropy I^1^ | 0.601/9/10 | | | | | 0.685/45/34 | | | | | | 0.692/16/12 | | | | 0.674/43/57 | |
| Entropy II^2^ | 0.574/10/9 | | | | | 0.573/24/55 | | | | | | 0.562/15/11 | | | | 0.572/41/65 | |
| Skewness I^1^ | 0.564/19/75 | | | | | 0.515/62/54 | | | | | | 0.503/30/33 | | | | 0.504/68/17 | |
| Skewness II^2^ | 0.505/66/74 | | | | | 0.620/20/39 | | | | | | 0.654/39/18 | | | | 0.595/63/38 | |
| **Color Correlation Features** | | | | | | | | | | | | | | | | | |
|  | | Red-green | | | Green-blue | | Blue-red | | | | Red-gray | | Green-gray | | | | Blue-gray |
| Lesion area only | | 0.680/-/31 | | | 0.632/-/6 | | 0.508/-/80 | | | | 0.673/-/49 | | 0.649/-/7 | | | | 0.625/-/69 |
| Whole cropped image | | 0.823/-/47 | | | 0.705/-/36 | | 0.773/-/53 | | | | 0.803/-/32 | | 0.821/-/37 | | | | 0.691/-/35 |
| **Color PCA Variance** | | | | | | | | | | | | | | | | | |
|  | | | PC1 | | | | | PC2 | | | | | | | PC3 | | |
| Variance I^1^ | | | 0.682/-/50 | | | | | 0.648/-/58 | | | | | | | 0.776/-/1 | | |
| Variance II^2^ | | | 0.504/-/82 | | | | | 0.712/-/30 | | | | | | | 0.818/-/8 | | |

Note: a/b/c represents the Az (univariate)/RFE ranking between 73 conventional features/RFE ranking between 91 features

SRE: short run emphasis. LRE: long run emphasis. LGRE: low gray-level run emphasis. HGRE: high gray-level run emphasis. SRLGE: short run low gray-level emphasis. SRHGE: short run high gray-level emphasis. LRLGE: long run low gray-level emphasis. LRHGE: long run high gray-level emphasis. GLNU: gray-level non-uniformity. RLNU: run length non-uniformity. RPC: run percentage. PC1, 2 and 3: the variance along the coordinates of the first, second and third principal components.

^1^Derived from the lesion area only.

^2^Derived from the whole cropped image.
